# Cell aging preserves cellular immortality in the presence of lethal levels of damage

**DOI:** 10.1371/journal.pbio.3000266

**Published:** 2019-05-23

**Authors:** Audrey Menegaz Proenca, Camilla Ulla Rang, Andrew Qiu, Chao Shi, Lin Chao

**Affiliations:** 1 Section of Ecology, Behavior and Evolution, Division of Biological Sciences, University of California, San Diego, La Jolla, California, United States of America; 2 CAPES Foundation, Ministry of Education of Brazil, Brasilia, Brazil; University of Washington, UNITED STATES

## Abstract

Cellular aging, a progressive functional decline driven by damage accumulation, often culminates in the mortality of a cell lineage. Certain lineages, however, are able to sustain long-lasting immortality, as prominently exemplified by stem cells. Here, we show that *Escherichia coli* cell lineages exhibit comparable patterns of mortality and immortality. Through single-cell microscopy and microfluidic techniques, we find that these patterns are explained by the dynamics of damage accumulation and asymmetric partitioning between daughter cells. At low damage accumulation rates, both aging and rejuvenating lineages retain immortality by reaching their respective states of physiological equilibrium. We show that both asymmetry and equilibrium are present in repair mutants lacking certain repair chaperones, suggesting that intact repair capacity is not essential for immortal proliferation. We show that this growth equilibrium, however, is displaced by extrinsic damage in a dosage-dependent response. Moreover, we demonstrate that aging lineages become mortal when damage accumulation rates surpass a threshold, whereas rejuvenating lineages within the same population remain immortal. Thus, the processes of damage accumulation and partitioning through asymmetric cell division are essential in the determination of proliferative mortality and immortality in bacterial populations. This study provides further evidence for the characterization of cellular aging as a general process, affecting prokaryotes and eukaryotes alike and according to similar evolutionary constraints.

## Abstract 2

A study of Escherichia coli shows that bacterial lineages maintain replicative immortality by reaching an equilibrium between aging and rejuvenation; when this equilibrium is disrupted, aging lineages cross their immortality threshold, becoming mortal, while rejuvenating lineages are favored by asymmetry and retain immortality within the same population.

## Introduction

Aging, or the progressive loss of function at the macromolecule, tissue, organ, or individual level, is largely driven by the deterioration of intracellular processes. Accordingly, the hallmarks of the aging phenotype—such as telomeric attrition, mitochondrial dysfunction, loss of proteostasis, and genomic instability—which have been well characterized by previous studies [1], reveal conserved genetic and biochemical pathways at the cellular level. Considering cellular aging as a baseline for the study of aging as a general process, we can summarize its mechanisms as the gradual intracellular accumulation of damage from various sources, along with a decreasing repair capacity. Furthermore, excessive damage accumulation within a cell lineage may lead to cellular senescence, in which individual cells cease replicating, and the lineage transitions to a mortal state [2–4].

The cellular aging process encompasses both multi- and unicellular organisms, such as yeast, diatoms, and even bacteria [5–8]. Due to the traditional view of unicellular prokaryotes as being functionally immortal, these organisms are often overlooked in the discussion of cellular aging. However, research in bacterial aging stands out for offering quantitative approaches to data collection and analysis, coupled with technical improvements on single-cell microscopy, which have detailed the aging phenotype and its progression. Although bacteria do not possess some of the eukaryotic aging targets, like telomeres and mitochondria, they are sensitive to stresses that induce nongenetic damage accumulation, such as oxidation and disruptions in protein folding [9,10]. Stressed bacteria accumulate misfolded proteins in the form of polar-localized aggregates [11–14], thus displaying loss of proteostasis. Repair occurs in a slow and energy-consuming fashion, in which chaperone proteins such as DnaK and ClpB mediate the disaggregation and unfolding of damaged proteins [10,12]. Additionally, the potential prokaryotic origin of mitochondria raises the possibility of regarding bacterial aging as a model for mitochondrial dysfunction, a noted hallmark of aging [1].

Besides aggregating and repairing damaged components, bacterial populations have developed another remarkable strategy to handle nongenetic damage. Experimental data from long-term microscopy of bacterial lineages revealed that, in the presence of intracellular damage, each cellular division produces 2 physiologically asymmetric daughters [8,11,15–17]. This asymmetry is generated because the damage harbored by the mother is biased toward the old cell pole [11,12], causing the daughter that inherits this pole—termed the old daughter—to age. Its sibling, on the other hand, rejuvenates through the inheritance of a lower damage load, being called the new daughter. Therefore, by partitioning damage with asymmetry, bacterial populations engage in a trade-off in which the fast growth of new daughters is sustained at the expense of the declining cellular function of old daughters. Mathematical models and computational simulations were developed to estimate the advantage of asymmetry, in contrast with a symmetric control population—a hypothetical scenario in which both daughters display equal physiology [18]. The models have shown that asymmetry is evolutionarily advantageous because it increases the variance of elongation rates, which in turn increases the efficiency of natural selection and the mean fitness of the lineage. Diverse studies are beginning to show that asymmetric partitioning is not unique to bacteria but an advantageous mechanism for the progression of cell lineages. In fact, this process was recently observed in neural, embryonic, and germline stem cells [19–21], in which damage allocation plays a central role in self-renewal capacity, fate determination, and somatic sequestration of damage.

A better understanding of how the key features of aging are interconnected requires the eventual development of conceptual and mathematical models that can integrate with experimental studies the growth and aging of individual organisms or cells. Unicellular systems, such as bacteria, satisfy all these requirements. Here, we show that the maintenance of proliferative immortality in *E*. *coli* lineages depends on the physiological equilibrium produced by contrasting damage accumulation and asymmetric partitioning. We demonstrate that unstressed lineages accumulate damage produced by standard respiration levels, subsequently partitioning this load with a level of asymmetry that allows for the dilution of damage within both new and old daughters. We show that *E*. *coli* mutants with decreased repair capacity also exhibit asymmetric new and old daughters, reaching distinct states of growth equilibrium. Furthermore, bacterial aging responds with a positive dosage relationship to an external damaging agent, which progressively disrupts proteostasis by increasing damage accumulation rates and disrupting asymmetry. With a sufficiently elevated stress level, the damage accumulation within old lineages surpasses their immortality threshold, leading these lineages to arrest division and become mortal. However, due to asymmetric partitioning, new lineages within the same population retain proliferative immortality. Our results show that the appropriate model and system can contribute to identifying the dynamics of mortality and immortality in the context of cellular aging.

## Results

### Functionally immortal bacterial lineages display damage accumulation and asymmetry

To determine whether bacterial lineages undergoing immortal proliferation displayed damage accumulation and partitioning dynamics, we cultured unstressed *E*. *coli* cells using microfluidic devices. We employed the “mother machine” design [22] containing series of 1.2-μm–wide growth wells at the bottom of which an old daughter remains trapped for the length of the experiment. Each well was connected in one end to large flow channels, constantly supplying fresh culture medium to maintain a healthy state for an extended time. Bacteria were loaded and tracked through time-lapse microscopy for 24 h in the absence of extrinsic damage. As an estimate of fitness, elongation rates and corresponding doubling time conversions were determined for each individual, along with its age, according to cell pole inheritance following division.

**Fig 1:**
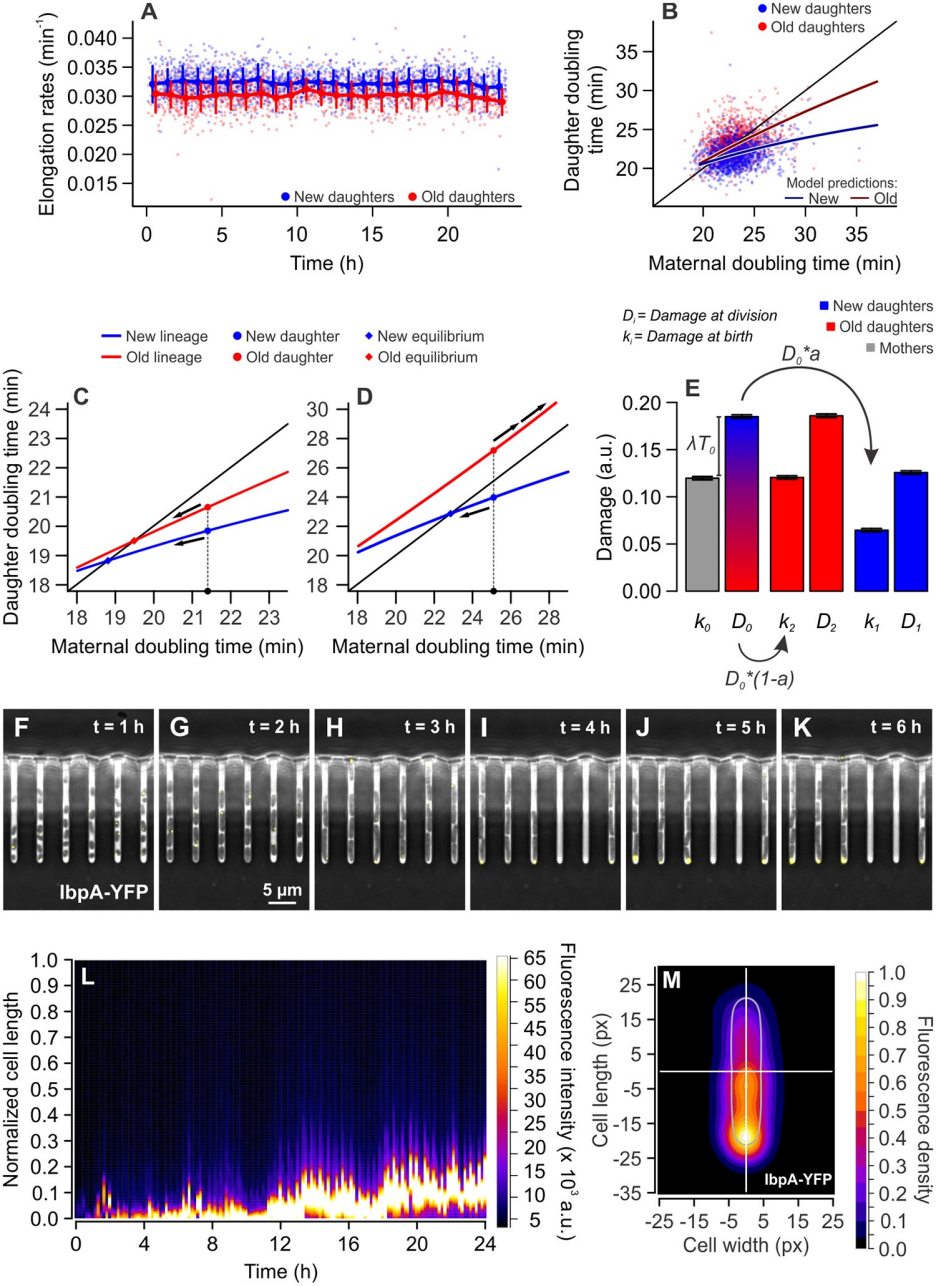
Maintenance of growth equilibrium and immortality through asymmetric damage partitioning. (A) New daughters (*n* = 1,782; 0.032 ± 0.0023 min^−1^ [mean ± SD]) elongated at significantly higher rates than old daughters (*n* = 1,285; 0.030 ± 0.0025 min^−1^ [mean ± SD]), a distinction that remained stable over several hours (one-tailed *t* test, *t* = 24.747, df = 2,612.5, *p* < 0.001). Binned data comprise mean ± SD. (B) The distinction between new and old daughters was also verified for the doubling times of sibling pairs (paired one-tailed *t* test, *t* = 24.716, df = 1,383, *p* < 0.001; S1 Data). The separation of new and old subpopulations, according to the estimation of growth parameters (see Materials and methods), was produced by the accumulation of damage at a rate λ = 0.0028 min^−1^, and the partitioning of such load with asymmetry *a* = 0.375. (C and D) Model predictions on cellular aging with Π = 18 min, *a* = 0.4. (C) With λ = 0.002 min^−1^, asymmetry produces a separation between new (blue) and old (red) subpopulations. The intersection of model predictions and the identity line creates equilibrium points, where *T*_*0*_ = *T*_*1*_ or *T*_*0*_ = *T*_*2*_, to which new or old daughters converge over generations (arrows). (D) With λ = 0.008 min^−1^, the old lineages are predicted to lose equilibrium and arrest division. New daughters, through constant rejuvenation, would retain replicative immortality at the same damage levels. (E) Damage load harbored by a mother and its daughters at the time of birth (*k*_*0*_, *k*_*1*_, *k*_*2*_) and division (*D*_*0*_, *D*_*1*_, *D*_*2*_; S7 Data). Applying the average growth parameters Π and λ to calculate *k*_*1*_ and *k*_*2*_, we verified that old daughters inherit larger damage loads than new daughters (paired one-tailed *t* test, *t* = 27.988, df = 1,244, *p* < 0.001) and also bear more damage at the time of division (paired one-tailed *t* test, *t* = 27.914, df = 1,244, *p* < 0.001). Bars represent mean ± SE. (F to K) Time lapse microscopy images showing the accumulation of misfolded proteins at the old cell poles over time. The small chaperone IbpA (yellow dots) colocalizes with damaged proteins, allowing the visualization of protein aggregates. (L) The fluorescence profile of an old lineage expressing IbpA-YFP shows that a protein aggregate develops over time, remaining trapped in the old pole over generations. Fluorescence profiles were measured every 10 min. See also S1 Fig for non-normalized length. (M) Combined IbpA-YFP fluorescence heatmap of 428 old daughters at the bottom of mother machine wells, imaged over 6 h. a.u., arbitrary units; IbpA, inclusion body protein A; YFP, yellow fluorescent protein.

Under such conditions, our previous studies have shown that new and old daughters display physiological asymmetry and long-term growth stability [17]. We confirmed these results in the present experiments, observing that new daughters displayed significantly faster elongation rates when compared with old daughters (Fig 1A), a distinction that remained constant over time. Moreover, comparing the maternal doubling time (hereby called *T*_*0*_) to that of its daughters (new = *T*_*1*_; old = *T*_*2*_) in a phase plane, a clear separation between new (21.79 ± 1.60 min) and old (23.23 ± 2.12 min [mean ± SD]) daughter subpopulations emerged (Fig 1B, S1 Fig, and S1 Data). In these conditions, the difference between *T*_*1*_ and *T*_*2*_ (*n* = 1,384 pairs) was significantly larger than zero (one-sample *t* test, *t* = 24.716, df = 1383, *p* < 0.001). These results suggest that old daughters in our populations are inheriting a larger damage load upon division, despite the absence of extrinsic damage in our growth conditions.

To quantify the possible damage accumulation and partitioning in these populations, we applied these results to a population genetics model on unicellular aging [23]. Because the accumulation of intrinsic damage positively correlates with increased doubling times, we can estimate maternal damage levels, the fraction inherited by each daughter upon division, and the resulting *T*_*1*_ and *T*_*2*_, reconstructing the progression of aging within a lineage. For this goal, we described cell lineage dynamics though 3 key parameters: Π, the doubling time of a damage-free cell; λ, the rate of damage accumulation within a single cell (0 to approximately 0.01 min^−1^); and *a*, the partitioning asymmetry, ranging from 0 (complete asymmetry) to 0.5 (symmetric division).

Our growth parameters revealed the presence of intrinsic damage and asymmetry in physiologically stable *E*. *coli* (S1 Table). Despite the ideal growth conditions provided by our microfluidic device, we found that bacterial populations displayed longer doubling times (22.34 ± 2.12 min [mean ± SD]) than predicted for damage-free cells (Π = 19.66 min; one-sample *t* test, *t* = 75.04, df = 3,482, *p* < 0.001). These longer doubling times were driven by damage accumulation—which occurred at an average rate λ = 0.0028 min^−1^—thus suggesting that metabolic processes in healthy cells may induce the retention of intrinsic damage. Finally, as suggested by the separation between *T*_*1*_ and *T*_*2*_ subpopulations, we verified that these damage loads were partitioned asymmetrically at division, with old daughters inheriting 63% of the maternal damage (*a* = 0.37).

### Damage accumulation and partitioning in stable growth equilibrium

The growth parameters Π, λ, and *a* can be used to predict doubling times *T*_*1*_ and *T*_*2*._ In Fig 1B, the solid lines show predicted doubling times for our average population parameters, thus showing the trend of new and old subpopulations. The crossing between these model lines and the identity line represents points of growth equilibrium, where *T*_*0*_ = *T*_*1*_ or *T*_*0*_ = *T*_*2*_ (Fig 1C). Asymmetric populations thus stabilize around 2 points simultaneously—one for new lineages and the other for the old, with cells continuously inheriting either pole remaining at equilibrium over generations [7,17,23]. Cell lineages in physiological equilibrium replicate indefinitely, thus remaining functionally immortal.

To confirm the long-term stability of new and old lineages in our experiments, we analyzed linear regressions between *T*_*0*_ and *T*_*1*_ or *T*_*2*_ as previously described [17] (S1 Fig). Bacterial lineages remain stable provided the existence of equilibrium points, which is satisfied by the intersection between each linear regression and the identity line (Fig 1C and S1 Fig). This intersection occurs when the slope of *T*_*1*_ or *T*_*2*_ lines is less than 1, which our data satisfy for both *T*_*1*_ (a = 0.246, *p* < 0.001) and *T*_*2*_ (a = 0.309, *p* < 0.001). In the stable environment of microfluidic devices, this equilibrium can still be disrupted by the stochasticity present in doubling times. This stochasticity can be described as random variables ξ_1_ acting on the slopes in *T*_*i*_ = *T*_*0*_ × (a + ξ_1_) + b, obtained in each generation from a Gaussian distribution with SD of σ_1_. Loss of equilibrium occurs when a^2^ + σ_1_^2^ ≥ 1 (see Materials and methods for details). We estimated σ_1_ for *T*_*1*_ and *T*_*2*_ lines by obtaining the deviations from slopes in (*T*_*i*_ − b) ÷ *T*_*0*_ = a + σ_1_. Both new (σ_1_ = 0.0657) and old (σ_1_ = 0.0876) lineages satisfied the stability requirement a^2^ + σ_1_^2^ < 1, with a^2^ + σ_1_^2^ = 0.0649 for *T*_*1*_ and a^2^ + σ_1_^2^ = 0.1032 for *T*_*2*_.

Besides the possibility of being disrupted by stochasticity, our aging model predicts that stable equilibrium can be disrupted by the accumulation of intrinsic damage [23]. Our parameters estimate that an increase in damage accumulation rates, from λ = 0.002 to 0.008 min^−1^, would progressively drive the equilibrium points toward longer doubling times. Because asymmetric partitioning produces higher doubling times in old daughters, a sufficiently intense λ would act as a differential mortality threshold, leading to division arrest—i.e., a state of mortality—in the old lineage, whereas new daughters remain immortal (Fig 1D).

To connect our observation of immortality and growth equilibrium to the internal dynamics of damage accumulation and partitioning, we estimated damage loads using our growth parameters. From experimental doubling times (S1 Data), we calculated the damage loads harbored by a mother, new daughters, and old daughters at the time of birth (*k*_*0*_, *k*_*1*_, *k*_*2*_) and division (*D*_*0*_, *D*_*1*_, *D*_*2*_; Fig 1E and S7 Data). It is important to note that this model considers the entirety of damage loads present in each cell, be it in aggregate or diffuse form. Each cell is born with a load *k*_*i*_, and accumulates λ × *T*_*i*_ over its lifetime, resulting in a load *D*_*i*_. We verified that *k*_*2*_ *> k*_*1*_, as expected from observed doubling times and asymmetry (paired one-tailed *t* test, *t* = 27.988, df = 1,244, *p* < 0.001). Furthermore, we compared each mother to its old daughter and verified that *k*_*2*_ = *k*_*0*_ (paired two-tailed *t* test, *t* = 0.373, df = 1,244, *p* = 0.709). This indicates that old lineages in a state of equilibrium, as observed in the mother machine, are born with a constant level of intrinsic damage. Consequently, the damage accumulated by a mother over its lifetime is equivalent to the load inherited by new daughters upon division, or λ × *T*_*0*_ = *k*_*1*_ (*t* = 0.367, df = 1,244, *p* = 0.714).

Taken together, our results suggest that unstressed bacterial populations accumulate intrinsic nongenetic damage. Every generation, new daughters inherit the damage a mother accumulated over its lifetime (*k*_*1*_ = *D*_*0*_ × *a* = λ × *T*_*0*_), whereas old daughters inherit the same amount the mother had at birth (*k*_*2*_ = *D*_*0*_ *× (1 − a) = k*_*0*_). These dynamics of damage accumulation and partitioning allow for a state of physiological equilibrium, in which old lineages display stable growth over time and retain proliferative immortality.

### Large protein aggregates become anchored at old cell poles

To visualize the biasing of damage loads toward old daughters in our microfluidic device, we cultured *E*. *coli* expressing the small chaperone inclusion body protein A (IbpA) fusioned to yellow fluorescent protein (YFP). IbpA-YFP was shown to colocalize with protein aggregates in bacterial cells [11], thus serving as a marker for the presence and position of nongenetic damage [24]. By culturing this strain in our microfluidic device, we observed the progressive accumulation of damage in the old poles of lineages in a state of equilibrium (Fig 1F–1M). We quantified the inheritance of IbpA-YFP fluorescent foci by following lineages over time, determining the subcellular localization of the aggregate and its partitioning upon division [25]. In over 194 cell divisions, we observed the appearance of 43 new fluorescent foci. Small foci first appeared in the center of a cell in 37.2% of the observations, diffusing freely throughout the bacteria (S1 Fig). However, as these aggregates accumulated more misfolded proteins, they quickly became anchored at the old poles (Fig 1L and 1M), resulting in the inheritance of fluorescent foci by old daughters in 80.4% of the observed division events (S1 Fig). It is important to note, however, that the YFP fusion might increase aggregation rates of the small chaperone IbpA [26], and unstressed cells likely harbor diffuse fluorescence and smaller foci rather than large aggregates. Nonetheless, the IbpA-YFP marker demonstrates the potential for asymmetric damage partitioning arising from the anchoring of protein aggregates at the old poles of old daughters over several generations.

### Asymmetry and immortal proliferation in protein repair mutants

**Fig 2:**
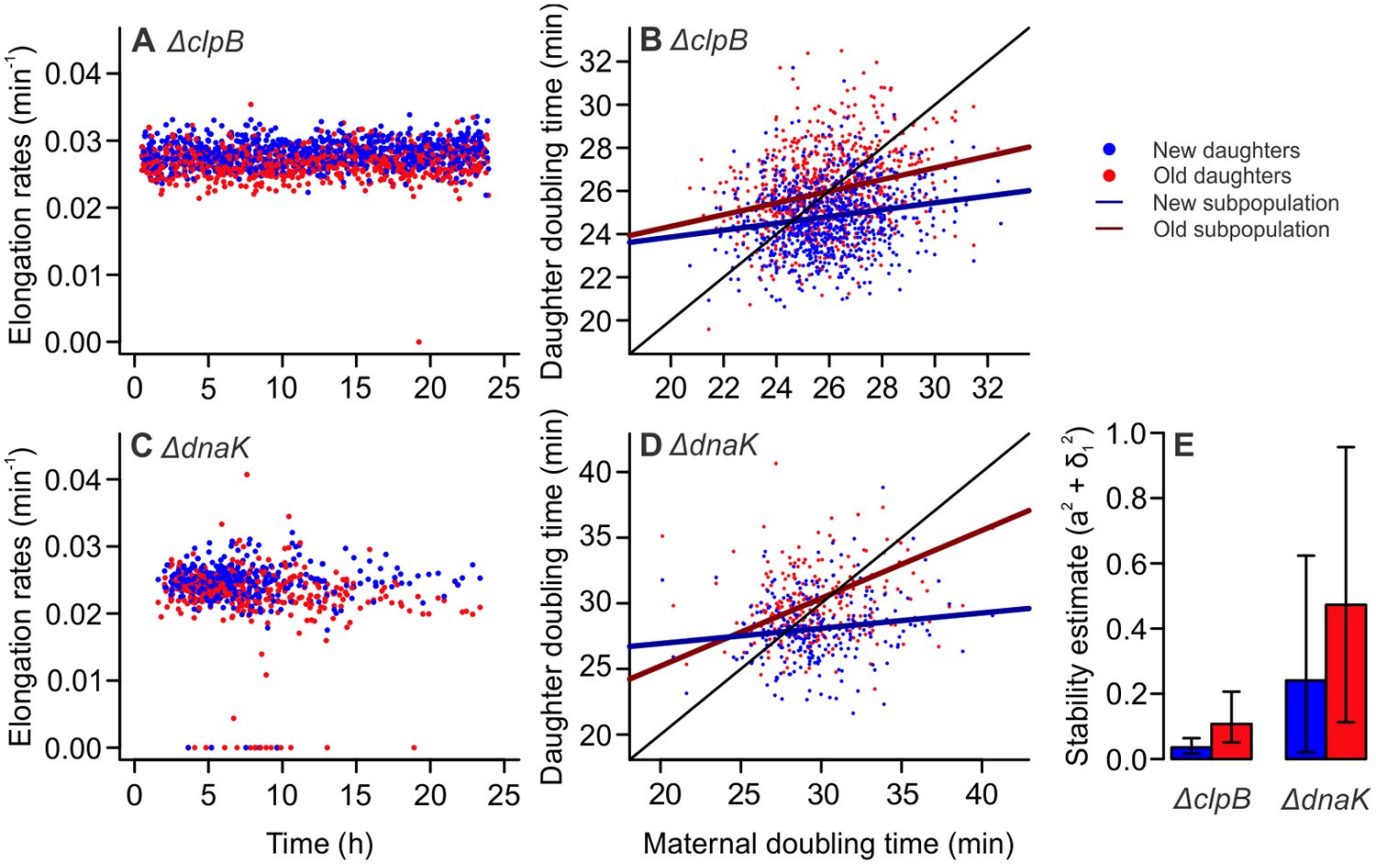
Equilibrium and asymmetry are present in repair mutants lacking ClpB or DnaK chaperones. (A) *ΔclpB* cells (*n* = 1,642; 0.027 ± 0.002 [mean ± SD]) exhibited stable elongation rates over time, suggesting these mutants might be in growth equilibrium. A single mortality event was observed. (B) *ΔclpB* mutants retained asymmetric doubling times (S2 Data). A two-way ANOVA indicated a significant effect of both *T*_*0*_ (F = 82.32, *p* < 0.001) and age (F = 178.07, *p* < 0.001) on doubling times, with interaction between factors (F = 5.66, *p* = 0.017). (C) *ΔdnaK* mutants (*n* = 786, 0.0236 ± 0.004) exhibited signs of stability loss, with several mortality events occurring throughout the experiment. (D) Similarly to *ΔclpB*, *ΔdnaK* mutants exhibited a separation between new and old subpopulations (S3 Data), with a two-way ANCOVA indicating a significant effect of *T*_*0*_ (F = 12.78, *p* < 0.001) and age (F = 16.89, *p* < 0.001) on doubling times, with interaction between factors (F = 5.11, *p* = 0.024). (E) A stability analysis performed on linear models from (B) and (D) revealed that both strains satisfy the stability requirement a^2^ + σ_1_^2^ < 1. Although mortality events were observed for *ΔdnaK* mutants, the strain remains mostly stable. Our bootstrap analysis (S7 Data) revealed a 1.45% probability of losing equilibrium for old lineages, and complete stability for new lineages (*x*^2^ = 129.86, df = 1, *p* < 0.001). Error bars: 95% CIs. CI, confidence interval; ClpB, chaperone protein ClpB; DnaK, chaperone protein DnaK.

Given the asymmetric damage partitioning in equilibrium lineages, we investigated the relevance of the protein repair machinery for the maintenance of proliferative immortality. For this, we employed *E*. *coli* single-gene knockout mutants lacking the chaperones ClpB or DnaK (Keio collection), which play a prominent role in the solubilization of protein aggregates [10,12,27]. We cultured these cells in mother machine devices as described above, screening bacterial lineages for asymmetric damage partitioning and physiological equilibrium. By following old lineages over time, we verified that both new and old *ΔclpB* daughters displayed constant elongation rates throughout the experiment (Fig 2A). We observed that *ΔclpB* mutants also displayed asymmetric doubling times, with new (24.81 ± 1.64 [mean ± SD]) daughters growing faster than their old (25.97 ± 1.87 [mean ± SD]) siblings (Fig 2B and S2 Data; paired one-tailed *t* test, *t* = 16.846, df = 770, *p* < 0.001). A distinct pattern emerged from the analysis of *ΔdnaK* mutants, with several mortality events occurring over time (Fig 2C). Nonetheless, a significant distinction between new (28.06 ± 2.49 [mean ± SD]) and old (30.20 ± 8.27 [mean ± SD]) daughter doubling times was observed in these cells (Fig 2D and S3 Data; paired one-tailed *t* test, *t* = 4.262, df = 266, *p* < 0.001).

To verify the stability of growth equilibrium in asymmetric *ΔclpB* and *ΔdnaK* populations, we analyzed the linear models presented in Fig 2B and 2D. To include mortality events in the analysis, doubling times were converted to elongation rates. We investigated whether the stochasticity present in the data could disrupt equilibrium stability in our populations, determining the noise acting on regression slopes as σ_1_ in (*T*_*i*_ − b) ÷ *T*_*0*_ = a + σ_1_. To generate 95% confidence intervals (CIs), we performed a 10,000-fold bootstrap on *T*_*0*_, *T*_*1*_, and *T*_*2*_ trios. We verified that *ΔclpB* mutants satisfied the stability requirement a^2^ + σ_1_^2^ < 1 (Fig 2E), thus remaining proliferatively immortal, for both new (a^2^ + σ_1_^2^ = 0.035 [0.016–0.064], mean [95% CI]) and old lineages (a^2^ + σ_1_^2^ = 0.107 [0.051–0.206]). For *ΔdnaK*, although new (a^2^ + σ_1_^2^ = 0.241 [0.020–0.624]) and old lineages (a^2^ + σ_1_^2^ = 0.473 [0.113–0.956]) satisfied the stability requirement, several mortality events were observed, with our bootstrap analysis suggesting a 1.45% probability of equilibrium loss (binomial test, *p* < 0.001) in *ΔdnaK* old lineages.

Taken together, these results suggest that repair chaperones ClpB and DnaK might have distinct roles in the maintenance of equilibrium stability. Although the decreased protein repair capacity in *ΔclpB* mutants still allowed for the stable proliferation of new and old daughters, old lineages in *ΔdnaK* mutants begin to show signs of stability loss. We therefore hypothesize that dynamics of damage accumulation may greatly impact proliferative immortality, with asymmetry determining a differential fate for new and old lineages.

### The transition from immortality to mortality is determined by damage accumulation and asymmetric partitioning

To investigate whether aging cell lineages would retain physiological equilibrium—therefore proliferative immortality—under increasing levels of damage accumulation, we cultured bacteria in the presence of extrinsic damage. We employed light excitation (490 nm wavelength), commonly used for green-fluorescent protein imaging, as a damaging agent known for inducing the production of reactive oxygen species and mitochondrial damage [16,28–30]. Bacteria were cultured in microfluidic devices and treated with variable lengths of light exposure, ranging from 70 ms to 3 s, administered every 2 min for up to 24 h. Each experiment was preceded by 24 h of control imaging in the absence of extrinsic damage.

**Fig 3:**
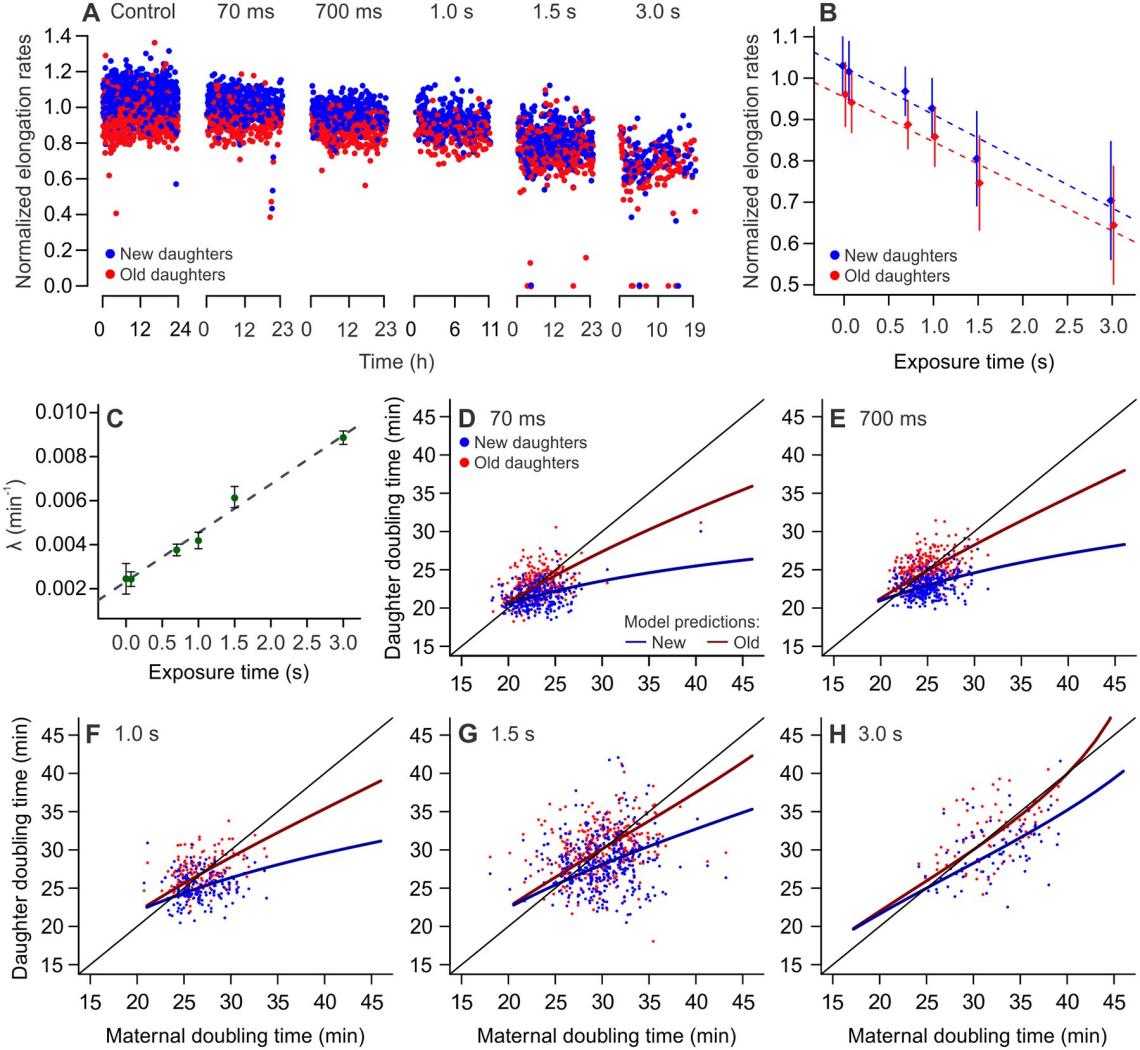
Damage accumulation decreases elongation rates and displaces growth equilibrium. All panels depict MG1655 wild-type *E*. *coli*. (A) Exposure to phototoxic damage led to decreasing elongation rates in all treatment levels (length of exposure, every 2 min: 70 ms, 700 ms, 1 s, 1.5 s, 3 s). (B) Both new and old daughters displayed slower growth in response to phototoxic damage (F = 9,272, *p* < 0.001), with new daughters growing faster than their old siblings in all cases (F = 1,505, *p* < 0.001). There was significant interaction between age and damage level in determining elongation rates (F = 2,384, *p* < 0.001; two-way ANCOVA). Data are represented as mean ± SD (S7 Data). (C) Linear correlation between length of phototoxic damage exposure and damage accumulation rates estimated for each treatment (*p* < 0.001, R^2^ = 0.98). Data are represented as mean ± 95% CI. (D to H) Distinct subpopulations of young and old daughters were observed in all treatment levels, with increasingly longer doubling time equilibria (S4 Data). At 3 s of exposure (λ = 0.009 min^−1^), the old lineages lie at the threshold of arresting proliferation.

Analyzing cell lineages over time, we observed a significant decrease in elongation rates on each exposure treatment relative to its control (Fig 3A and S2 Table; unpaired two-sample *t* tests; *p* < 0.001). The treatments revealed a significant effect of both age and light exposure on individual elongation rates, with new daughters maintaining significantly faster growth than their old siblings in all cases (Fig 3B). A similar effect was observed for the impact of light exposure and age on damage inherited at birth (S2 Fig), showing that the overall damage inheritance increased with treatment. We determined the growth parameters of each treatment (S3 Table), verifying that λ increased linearly with the length of exposure to light excitation (Fig 3C). This demonstrates that the rates of extrinsic damage infliction correlate linearly with the rate of intracellular damage accumulation for our experimental design. From the growth parameters Π, λ, and *a*, we calculated the estimated doubling times of new and old lineages, as well as the predicted doubling time equilibria for each treatment level (Fig 3D to 3H, S4 Data). Our results showed a separation between new and old daughter subpopulations in all cases (S4 Table).

**Fig 4:**
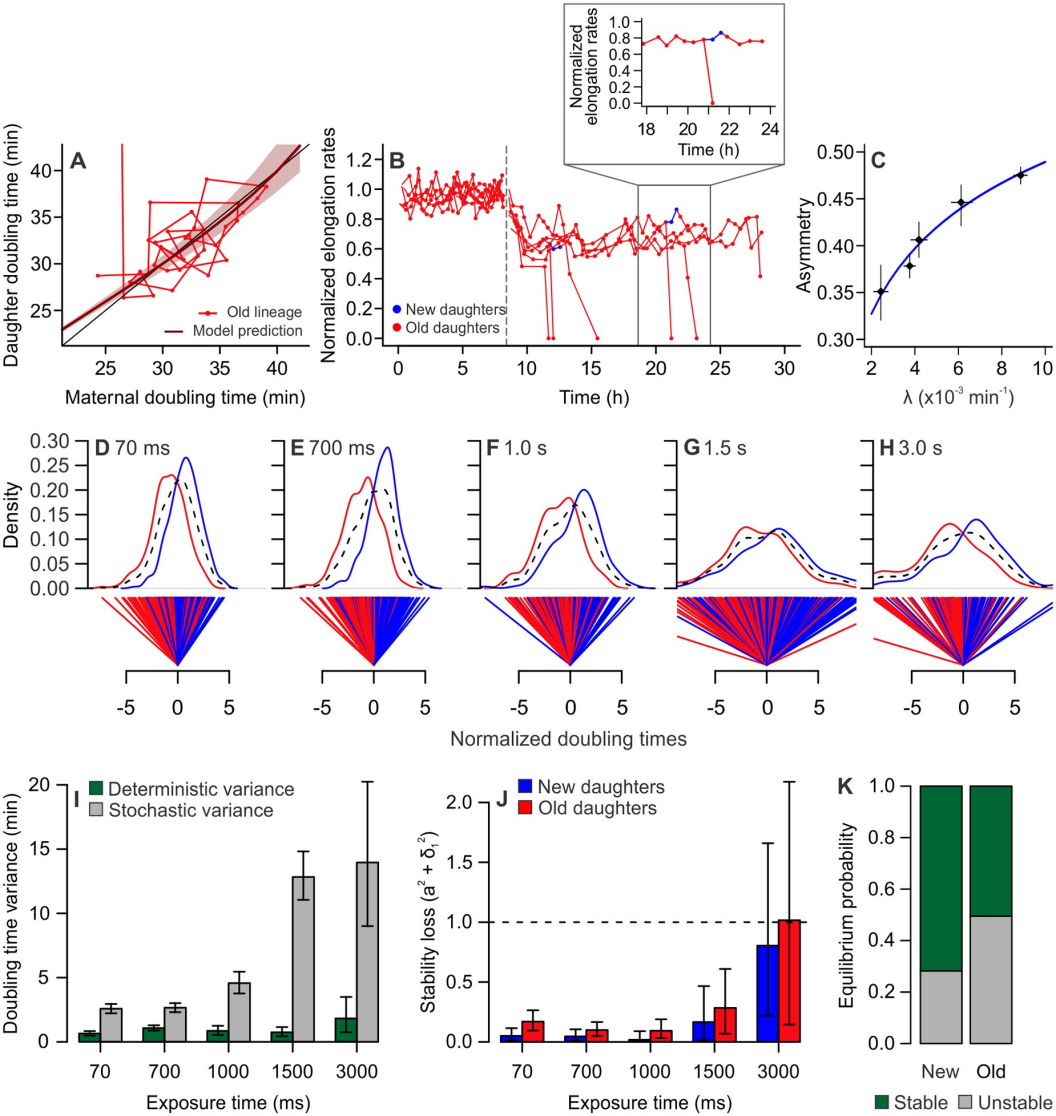
Damage accumulation leads to mortality and disrupted asymmetry. All panels depict MG1655 wild-type *E*. *coli*. (A) Doubling times of an old lineage as it accumulates damage at λ = 0.009 min^−1^_,_ induced by 3 s of light exposure. The line representing model predictions (dark red) approaches identity (black), indicating that these cells do not reach equilibrium. After a few generations, the last daughter in the lineage arrests growth, which equals an infinite doubling time, which represents the crossing of a mortality threshold for the lineage. (B) Elongation rates of old lineages, showing the transition from control imaging (0–8.6 h) to the infliction of 3 s of light exposure every 2 min. All cells exhibited lower growth rates, culminating in division arrest for old daughters. New daughters outlived their old siblings by at least one generation and were sometimes able to generate a new lineage in the growth wells (shown in blue and expanded in the detail). All values were normalized by the average control elongation rates. (C) Increasing damage accumulation rates disrupted asymmetric partitioning, as shown by the asymmetry coefficient approaching 0.5 (*a* = 0.1007 × ln(λ) + 0.95, *p* < 0.001). Points represent average growth parameters and 95% CIs (S7 Data). (D–H) Distributions of new (blue) and old (red) daughter doubling times were normalized around a symmetric midpoint. Whereas the combined population has a distribution centered at zero (dashed black lines), new and old subpopulations split into 2 separate distributions. The distance between the averages of these distributions expresses the doubling-time variance produced by deterministic physiological asymmetry. The average variance of new and old distributions around their own means, on the other hand, represents the variance produced by stochasticity. (I) Deterministic and stochastic portions of the variance from (D–H) were summarized for increasing light exposure, showing an increase in stochasticity. Error bars represent 95% CIs. (J) Our stability analysis indicated that new and old daughters remained in stable equilibrium until exposed to 3 s of phototoxic stress. At 3 s, old daughters no longer satisfy the stability requirement (a^2^ + σ_1_^2^ = 1.016), thus transitioning to a mortal state. Error bars represent 95% CIs (S7 Data). (K) At 3 s of exposure, old lineages displayed a 50.24% probability of losing equilibrium, whereas new lineages exhibited only a 28.10% probability of mortality (test for equality of proportions, *x*^2^ = 1027.7, df = 1, *p* < 0.001). CI, confidence interval.

The increasing induction of damage accumulation led to the stabilization of new and old lineages at equilibria with progressively longer doubling times. Extreme damage levels caused the old lineage equilibrium to approach infinite doubling times (Fig 4A and 4B) with 3 s of exposure, meaning that old daughters undergo division arrest, suggesting a damage accumulation rate of 0.009 min^−1^ as the threshold at which aging lineages transition to mortality. Fewer mortality events were observed in new daughters, indicating that new lineages might remain proliferative under the same conditions. We observed that the difference between damage loads at birth (*k*_*2*_ and *k*_*1*_) was significantly reduced at 3 s of exposure, when compared to control conditions (S2 Fig; two-tailed *t* test, *t* = 2.805, df = 80.995, *p* = 0.0063). This outcome was surprising, because one of the advantages of asymmetric damage partitioning in bacterial populations is the ability to endure higher levels of damage [18]. Therefore, we expected to find that populations exhibiting large damage accumulation rates should display greater asymmetry.

Our experiments, nonetheless, revealed a consistent pattern of diminishing asymmetry with the infliction of light excitation. Although all populations remained asymmetrical, with a maximum *a* = 0.47, the asymmetry coefficient approached 0.5 as λ increased (Fig 4C). A possible driver of increasing symmetry would be the fast accumulation of new damaged components, as expressed by increasing λ, surpassing the rate at which such components aggregate. As a result, more damage would be partitioned as diffused rather than polar anchored molecules at the time of division [18], leading to an increase in stochastically partitioned damage. To investigate this hypothesis, we tested whether the doubling time variance produced by stochasticity increased with light exposure. We first normalized the doubling times of each sibling pair around the expected values for symmetric cells (Fig 4D–4H) for each treatment level. This normalization removes the variance produced by noise in maternal growth. Because new and old daughters in our populations are physiologically distinct, 2 distributions arise from the normalized data. The distance (D) between these distributions is produced by asymmetry, which defines the variance explained by deterministic factors as D^2^ ÷ 4 (see Materials and methods and [18] for details). The average variance of new and old distributions (V_N_ + V_O_) ÷ 2, on the other hand, represents the doubling time variance explained by stochasticity. The estimates of deterministic and stochastic variance were summarized in Fig 4I and S5 Table. With higher levels of light excitation, we observed an increase in the variance explained by stochasticity, whereas the deterministic variance remained nearly constant. These results indicate that, although the deterministic physiological distinction between new and old daughters remains present, the perceived asymmetry between these lineages is attenuated by stochasticity as extrinsic damage levels increase. It is important to observe that, although our results depict the effect of constant damage exposure, other interesting outcomes could arise from transient damage pulses.

To determine whether new and old lineages remained in equilibrium despite the presence of large stochasticity, we performed a stability analysis on elongation rates. We followed the same principles described in Fig 2 and S1 Fig, in which a linear regression between *T*_*0*_ and *T*_*1*_ or *T*_*0*_ and *T*_*2*_ is evaluated for its stability in crossing the identity lines. The maintenance of this crossing, which acts as an equilibrium attractor, determines that these lineages display stable growth over time, thus retaining immortal proliferation. In the presence of stochasticity acting on the regression slope, the condition a^2^ + σ_1_^2^ < 1 must be satisfied for the retention of equilibrium. We estimated the slopes and σ_1_ values for each light exposure treatment (Fig 4J), performing a bootstrap analysis to obtain CIs. The stability condition in our experiments was reached by all lineages until 3 s of light exposure. At 3 s of exposure, or λ = 0.009 min^−1^, old lineages reached their mortality threshold and became unstable, resulting in the mortality events observed in Fig 4A and 4B. All new lineages in our experiments remained stable. However, because the CIs in Fig 4J indicated a chance of new lineages also losing stability at 3 s, we investigated the probability of retaining immortal proliferation in Fig 4K. Our analysis revealed that old lineages exhibited a significantly higher probability of losing equilibrium (50.24%) than new lineages (28.10%, x^2^ = 1027.7, df = 1, *p* < 0.001), thus indicating that asymmetric damage partitioning leads to differential maintenance of immortality in new and old lineages within the same population. These results suggest that, despite the decrease in asymmetric partitioning, new daughters are able to endure higher levels of damage while remaining functionally immortal.

### Old lineages transition from immortality to mortality under extrinsic stress

**Fig 5:**
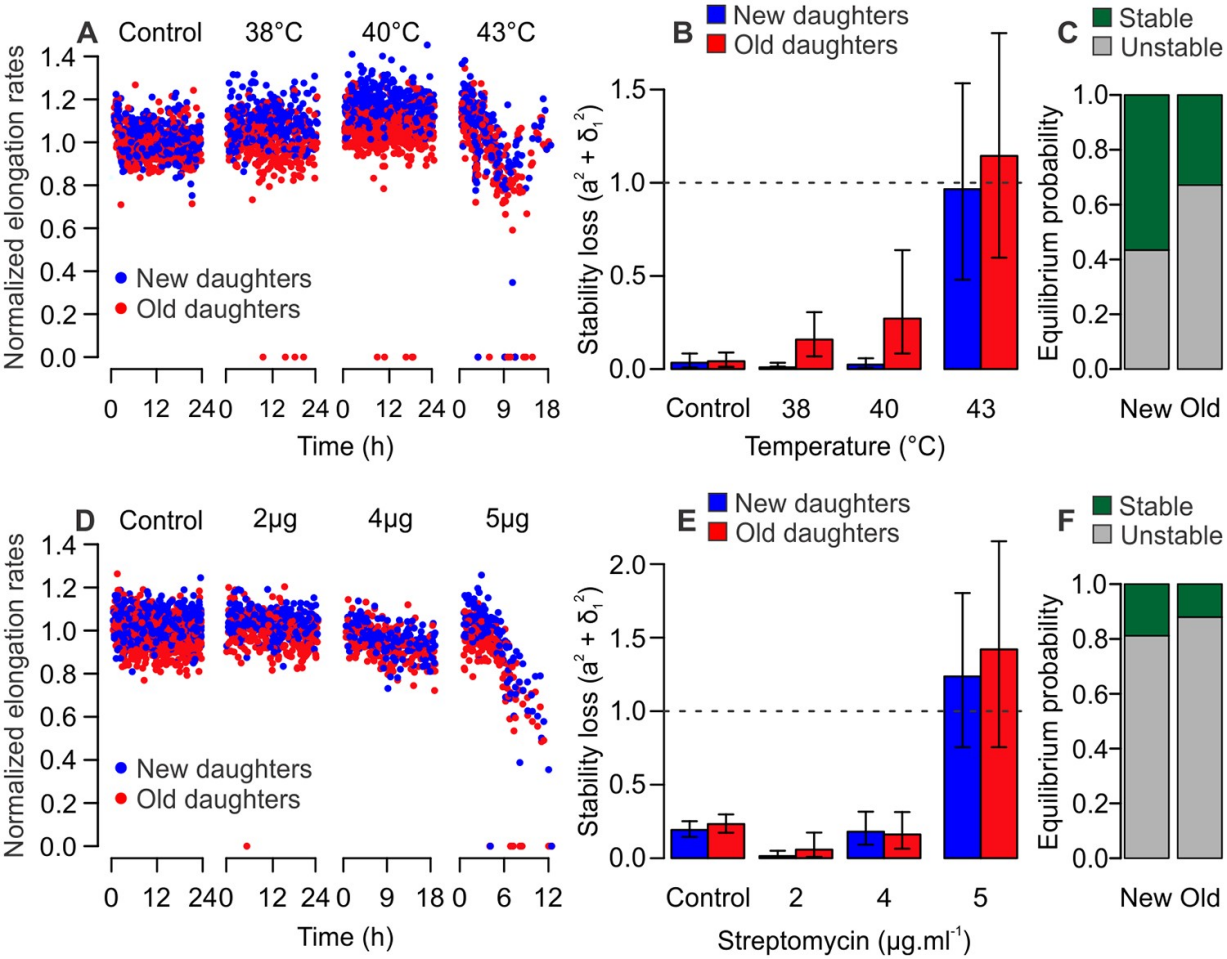
Old lineages are more likely to reach mortality when exposed to heat or streptomycin. All panels depict MG1655 wild-type *E*. *coli*. (A) Elongation rates over time for populations exposed to control temperatures, 38 °C, 40 °C, or 43 °C heat stress (*n* = 875, 535, 782, and 380 cells; S5 Data). A few mortality events were observed for 38 °C and 40°C, with elongation rates declining at 43 °C. (B) At 38 °C and 40 °C, both new and old lineages satisfied the stability requirement a^2^ + σ_1_^2^ < 1. At 43 °C, new lineages in our experiment remained stable, whereas old lineages lost equilibrium. (C) At 43 °C, old lineages displayed a significantly higher probability (67.1%) of losing equilibrium than new lineages (43.4%, x^2^ = 1136, df = 1, *p* < 0.001; S7 Data). (D) Populations exposed to 0, 2, 4, or 5 μg/ml streptomycin (*n* = 1,322, 453, 337, and 292 cells; S6 Data) showed declining elongation rates over time. (E) Both lineages remained in stable equilibrium for 2 and 4 μg/ml streptomycin; however, new (a^2^ + σ_1_^2^ = 1.236) and old (a^2^ + σ_1_^2^ = 1.420) lineages lost stability at 5 μg/ml. (F) Although both lineages displayed a large probability of transitioning to mortality, old lineages (88.0%) had a higher chance of losing equilibrium than new lineages (81.2%, x^2^ = 177.13, df = 1, *p* < 0.001; S7 Data) at 5 μg/ml streptomycin. (B and E) Error bars represent 95% CIs. CI, confidence interval.

To determine whether the differential mortality of new and old daughters could be translated to other damage sources, we repeated our experiments replacing light exposure with heat stress (Fig 5A–5C) or streptomycin (Fig 5D–5F) as damaging agents. We exposed cells growing in the mother machine to 38 °C, 40 °C, and 43 °C, as heat exposure can lead to the accumulation of misfolded proteins and senescence [12,31]. We observed an increase in mortality events at 38 °C and 40 °C, although elongation rates remained constant over time (Fig 5A and 5B, S4 Table, and S5 Data). At 43 °C, however, elongation rates declined and old lineages lost stability (a^2^ + σ_1_^2^ = 1.144), whereas new lineages remained in equilibrium (a^2^ + σ_1_^2^ = 0.965). Our bootstrap analysis suggested that old lineages had a higher probability of transitioning to mortality (67.1%) than new lineages (43.4%, x^2^ = 1136, df = 1, *p* < 0.001; Fig 5C). We verified a similar outcome for populations exposed to 2, 4, or 5 μg/ml of streptomycin, which has been shown to induce protein misfolding in *E*. *coli* [14,16,32]. Although new and old lineages remained stable at 2 and 4 μg/ml (Fig 5D and 5E, S4 Table and S6 Data), both lineages lost stability at 5 μg/ml. Still, our analysis detected a differential probability of crossing the mortality threshold, with new lineages displaying a lower chance (81.2%) of becoming mortal than old lineages (88.0%, x^2^ = 177.13, df = 1, *p* < 0.001; Fig 5F). Taken together, these results suggest that the asymmetric partitioning of damage leads to a differential transitioning from immortality to mortality in stressed bacterial populations. The asymmetric allocation of nongenetic damage, whether inflicted by light exposure, heat, or streptomycin, leads to higher mortality in old lineages while allowing the immortality of new lineages within the same population. Therefore, these observations offer cellular aging as a model for both the maintenance of continuous replication, as in stem cells, and the loss of proliferative capacity due to cellular aging.

## Discussion

Individuals age by progressively accumulating damage over their life span, leading to loss of function late in life [33,34]. Because biological organisms are composed of individual cells, the process of cellular aging represents a baseline for understanding the general principles of aging and its phenotypic manifestations. Cellular aging comprises the dynamics of intracellular damage accumulation and partitioning, whose manipulation and quantification becomes possible in unicellular systems, such as bacteria. Bacterial populations display phenotypic variation arising from asymmetric cell divisions, an evolutionarily advantageous strategy for increasing the efficiency of natural selection [18]. Previous studies have shown that, as a consequence of asymmetric cellular divisions, aging and rejuvenating bacterial lineages stabilize at distinct states of physiological equilibrium [7,17,23]. While in equilibrium, these lineages remain functionally immortal. Here, we showed that this state of equilibrium is maintained by the balance between damage accumulation and asymmetric partitioning. Unstressed bacterial lineages, despite their immortal proliferation and constant environment, accumulated damage derived from standard metabolic rates and partitioned around 63% of their damage load towards old daughters. Repair mutants lacking the repair chaperones still retained asymmetric partitioning and the ability to reach equilibrium, supporting the notion that asymmetry contributes toward proliferative immortality in lineages that must rejuvenate constantly. This is the case of stem cells, which were recently shown to asymmetrically segregate damaged components and proteins targeted for degradation [19–21].

Although stem cell lineages rejuvenate at every division, their proliferation reaches exhaustion in old individuals [1]. Stem cells from old mice were also shown to have a disrupted diffusion barrier [20], which renders division more symmetric by causing the stem sibling to inherit damaged components. Our results suggest that a similar phenomenon takes place in bacterial lineages. We expected to find a larger asymmetry between daughter cells produced under high levels of extrinsic damage, but instead the treatments caused a disruption in efficient asymmetric partitioning and increased stochasticity. Because bacterial asymmetry depends on the allocation of misfolded proteins to old cell poles, cells exposed to high levels of stress might be failing to sequester their damaged components. It is possible that old poles become saturated with damage, causing aggregates of misfolded proteins to be randomly deposited in the new pole or along the cell. Another possibility is that high damage accumulation rates interfere with the repair machinery, composed of chaperones that colocalize with damage and are responsible for maintaining proteostasis [10,12,27].

The fact that asymmetry was disrupted by damage accumulation did not prevent new and old lineages from undergoing strikingly distinct paths under intense levels of extrinsic damage. When extreme damage accumulation rates are induced, new lineages display increased doubling times but remain in equilibrium. Old lineages, however, undergo division arrest as a consequence of inheriting larger damage loads, satisfying the classical pattern of cellular aging. In this scenario, old daughters have reached the mortality threshold, whereas new daughters remain functionally immortal. Asymmetry therefore allows for the coexistence of two distinct physiological states in a clonal cell population. If this mechanism can be extrapolated to cells within somatic tissues, asymmetric damage segregation could offer an explanation for the simultaneous occurrence of senescent and proliferative cells in aging tissues [2,4]. Because asymmetry dictates mortality or immortality in sibling cells, it may also relate to the processes of cellular differentiation and fate determination.

Addressing the study of aging from a cellular perspective, our findings showed that bacterial systems can provide an integrative view of the general principles driving the aging phenotype. From a simple cellular system, we can quantify the dynamics of damage accumulation and partitioning along generations. Asymmetric partitioning of damage drives cell populations to reach a stable equilibrium, in which the aging of a lineage enables the continued rejuvenation of another. Moreover, even when old lineages cross the threshold and become mortal, asymmetry allows the survival of new daughters and ensures the continuity of the population. Applying this framework to the aging research may largely contribute to the understanding of an evolutionarily conserved basis for the progressive functional decline experienced by prokaryotes and eukaryotes alike.

## Materials and methods

### Bacterial strains and growth conditions

Experiments were performed with K-12 *E*. *coli* wild-type strain MG1655 for the determination of damage accumulation in immortally proliferating bacteria and for experiments on the disruption of growth equilibrium. The visualization of protein aggregates was performed with MG1655 *E*. *coli* expressing YFP bound to the small heat-shock protein IbpA, constructed according to Rang and colleagues [24] from the construct *IbpA-yfp-Cm*^*r*^ kindly provided by Ariel B. Lindner (INSERM, France) [11]. Repair mutants were screened for asymmetry and equilibrium using *E*. *coli* BW25113 *ΔclpB* (CGSC #11763) and *ΔdnaK* (CGSC #8342) from the Keio knockout collection [35]. The antibiotic resistance marker was not removed from these strains. For all experiments, cultures were inoculated in lysogeny broth (LB broth; per liter: 10 g tryptone, 5 g yeast extract, 5 g NaCl; Sigma-Aldrich) and grown overnight at 37 °C with agitation. The culture medium was supplemented with 0.075% Tween 20 upon inoculation within microfluidic devices, which prevents the formation of biofilms in the flow channels.

### Microfluidic device design and fabrication

The device used in this study was based on the mother machine design by Wang and colleagues (2010), subsequently modified by Ryan Johnson (University of California, San Diego) for the addition of more growth wells. This device included 16 parallel flow channels containing 2,000 growth wells (1.25 × 30 × 1 μm) each. Polydimethylsiloxane (PDMS; Kit Sylgard 184, VRW International, California) microfluidic chips were fabricated from master silicon wafers used as negative molds, provided by the Ryan Johnson and the Jeff Hasty Lab (University of California, San Diego). PDMS chips fabricated through soft lithography yielded 12 devices per process and were attached to 24 × 40 mm coverslips through a covalent bond. Previous control experiments have shown that the asymmetry observed in mother machine devices is not produced by starvation [17,22] and that wide (>1.0 μm) growth channels can produce cells with faster growth rates than liquid cultures [36].

### Cell loading and experimental conditions

Cultures were grown overnight in LB medium and centrifuged for 2 min at 5,300*g*. The supernatant medium was subsequently discarded, and the pellet was resuspended in 50 μL of medium supplemented with Tween 20. Prior to loading, microfluidic devices were placed in a vacuum chamber for 10 to 15 min. Bacteria were loaded by placing a droplet of concentrated culture over the loading port, posteriorly used as an outlet during the experiment, and a droplet of sterile medium over the opposite port. Once all channels were properly filled, bacteria were pushed into the growth traps by centrifuging the device at 1,410*g* for 7 min. Input and output 60 ml syringes were connected to the ports for a continuous supply of growth medium throughout the experiment. The device was incubated at 37 °C during imaging. When required, extrinsic damage was induced by fluorescent light exposure (490 nm wavelength) using a FITC filter, set at 25% strength. The length of exposure to light excitation ranged from 70 ms to 3 s, applied in 2-min intervals. Damage induced by heat stress was produced by increasing the incubation temperature in the microscope chamber to 38 °C, 40 °C, or 43 °C, which was monitored in real time. Extrinsic damage induced by subinhibitory streptomycin concentrations was introduced by adding 2, 4, or 5 μg/ml of antibiotic to the growth medium. Each of these experiments was preceded by a 24-h control imaging of the same bacterial lineages.

### Time-lapse image acquisition

Cell movies were collected by a Nikon Eclipse Ti-S microscope, with imaging intervals controlled by NIS-Elements AR software. Phase images were collected in 2-min intervals during the entire length of mother machine experiments, immediately followed by the acquisition of FITC pictures when required. For heat or streptomycin stress experiments, no FITC imaging was used.

### Quantification of bacterial growth

Images were analyzed with the free software ImageJ (NIH, https://imagej.nih.gov/ij), recording cell coordinates as regions of interest (ROIs) and cell names as indicatives of lineage and cell pole inheritance. Cell lengths were determined immediately before and after each division, and time of division was recorded. Elongation rates (r) and doubling times (ln(2) ÷ r) were calculated from the data, and the resulting tables were entered in an R program to determine maternity, sibling pairs, and lineage trees. To ensure that the measurements were unbiased, we performed blind data collections in which elongation rates were recorded without knowledge of pole inheritance. The ImageJ plugin MicrobeJ was used for the creation of fluorescence profiles and heatmaps [37].

Data presented in Proenca and colleagues [17] for verifying the stability of the old lineage equilibrium attractor were included in our control data from phase imaging, accompanied by new control experiments performed for this study. These experiments provided the necessary baseline for our aging model parameters.

### Statistical analysis

Statistical analysis was performed using R version 3.4.1. *p* < 0.05 was considered statistically significant. Because distinct microfluidic devices yield small yet robust measurement differences, elongation rates and doubling times were normalized by population means when comparing data from different experiments. Data from phase imaging, our controls, were normalized by their respective averages. Normalized data for all replicates were pooled and compared by one- or two-sample *t* tests, as reported. Data from light exposure, heat, or streptomycin stress imaging were normalized by the respective control experiment mean elongation rates. Raw data corresponding to these normalizations were presented in phase planes for individual populations. Statistical parameters were reported as mean ± SD, or as mean ± 95% CIs for growth parameters, as indicated in the text. Sample sizes (cells, sibling pairs, or replicates, as informed) are indicated along with reports of statistical analyses.

### Cellular aging model

This population genetics model determines the role of asymmetric partitioning of damage upon cell division as a mechanism of survival in the presence of damage [23]. It was developed for bacterial populations, assuming that cells must build up an intracellular product to a checkpoint before dividing. Based on the rate with which a bacterium accumulates damage during its lifetime (λ) and the doubling time of fittest cell (Π), the damage load received at birth (*k*_*0*_) by a mother bacterium can be determined from its doubling time (*T*_*0*_) as

k0=1-(λ2)T0-ΠT0.

The load received at birth (*k*_*0*_), along with the amount accumulated in its lifetime (λ*T*_*0*_), is the damage a bacterium will segregate to its daughters according to the asymmetry coefficient (*a*), ranging from 0 (complete asymmetry) to 0.5 (symmetry):

$$k_{1}=(k_{0}+\lambda T_{0})a;$$

$$k_{2}=(k_{0}+\lambda T_{0})(1-a).$$

This asymmetric inheritance will affect the doubling times of the offspring, causing old daughters, which receive the higher load (*k*_*2*_), to slow down compared with their young siblings. The doubling times of each daughter, *T*_*1*_ and *T*_*2*_, are given by

$$T_{1}=\frac{(1-k_{1})-\sqrt{{\left(1-k_{1}\right)}^{2}-2\Pi \lambda }}{\lambda };$$

$$T_{2}=\frac{(1-k_{2})-\sqrt{{\left(1-k_{2}\right)}^{2}-2\Pi \lambda }}{\lambda }.$$

Estimates of doubling time equilibrium were determined as the points where prediction lines cross the identity, which can be calculated as

$$\alpha =\frac{a}{1-a};$$

T^1=1-1-4Πλ(12+α)2λ(12+α);

T^2=1-1-4Πλ(12+1α)2λ(12+1α).

### Estimation of growth parameters

The data collected for doubling times of trios composed by a mother bacterium and its two daughters were entered in the model to determine growth parameters. The doubling times of the daughters (*T*_*1*_ and *T*_*2*_) were estimated from a known maternal doubling time, using varying values of Π, λ, and *a*, and compared with the observed doubling times. Optimal parameters were those that provided the least mean squared difference between expected and observed doubling times. An independent combination of Π, λ, and *a* was estimated for each control experiment, whereas only λ and *a* were estimated for light treatment experiments (because Π is the baseline doubling time, it was provided by the respective control parameter). Nonsensical parameters were excluded based on previous knowledge of the model, such as the impossibility of *a* being either negative or larger than 1, or Π being larger than any observed doubling time. To obtain the 95% CIs for each parameter, the average of results was entered in a bootstrapped estimate of parameter combinations, repeated 10,000 times with resampling of the observed mother and daughter trios.

### Estimation of deterministic and stochastic variance components

Doubling time variances were analyzed for deterministic and stochastic components according to Chao and colleagues [18]. For each *T*_*0*_, *T*_*1*_, *T*_*2*_ trio, the doubling time of a hypothetical symmetric daughter was estimated based on Π, λ, and *T*_*0*_. Daughter doubling times were normalized by subtracting *T*_*1*_ and *T*_*2*_ from the symmetric daughter estimate, thus centering the mean distribution around zero. The distance (D) between the means of normalized *T*_*1*_ and *T*_*2*_ was estimated, as well as the variance of new (V_N_) and old (V_O_) distributions. The total variance (V_T_) in the population corresponds to V_T_ = (V_N_ + V_O_) ÷ 2 + (D^2^ ÷ 4). In this equation, D^2^ ÷ 4 represents the variance produced by deterministic asymmetry. (V_N_ + V_O_) ÷ 2 represents the unexplained variance, produced by stochastic sources.

### Equilibrium stability analysis

*T*_*0*_, *T*_*1*,_ *T*_*2*_ trios from phase planes were used to determine the stability of growth equilibria for new and old lineages. When mortality events were common (stressed populations), elongation rates were used instead of doubling times. New and old lineages were analyzed separately according to linear regressions between *T*_*0*_ and *T*_*1*_ or *T*_*2*_. The effective slope for each mother-daughter pair was determined as (*T*_*2*_ − b) ÷ *T*_*0*_ = a + ξ_1_, where ξ_1_ represents a random variable drawn from a Gaussian distribution each generation. The SD of the ξ_1_ distribution is given by σ_1_. As described by Proenca and colleagues [17], a point of equilibrium where the regression and identity lines intersect exists as long as the condition a^2^ + σ_1_^2^ < 1 is satisfied. Values of a^2^ + σ_1_^2^ were estimated for new and old daughters of experimental populations and reported as bar plots for all damaging conditions. This estimate was repeated as a 10,000-fold bootstrap of *T*_*0*_, *T*_*1*,_ *T*_*2*_ trios for the determination of 95% CIs and equilibrium probabilities.

## Supporting information

S1 Fig Stability and protein aggregation in unstressed populations. Growth stability in new and old lineages can be expressed by linear regressions between *T*_*0*_ and *T*_*1*_ (A, solid blue line) or *T*_*2*_ (B, solid red line). The intersect between regression lines and the identity line represents a point of stable equilibrium where doubling times converge. Due to the doubling time variance produced by stochasticity acting on the slopes (σ_1_), given by *T*_*i*_ = *T*_*0*_ × (a + σ_1_) + b, equilibria might be disrupted when a^2^ + σ_1_^2^ ≥ 1. Dashed lines in (A) and (B) represent the maximum variation in regression lines obtained by the parameter σ_1_ acting on the slopes of our data, demonstrating that new and old lineages retain equilibrium in the presence of stochasticity. (C) Fluorescence profiles obtained in 10-min intervals for an old lineage, showing the anchoring of protein aggregates (IbpA-YFP) in the old pole over time. (D) Over the course of 194 cell divisions observed over 24-h imaging, we verified the first appearance of 43 protein aggregates. The cellular localization of these new fluorescent foci showed no bias for old poles. (E) The partitioning of new protein aggregates upon division showed higher inheritance by new daughters (62.79% of cell divisions, *n* = 43, *χ*^2^ = 4.651, df = 1, *p* = 0.031). However, old daughters inherited the majority of recurring aggregates (97.90% of cell divisions, *n* = 143, *χ*^2^ = 258.69, df = 1, *p* < 0.001) as these became anchored to old cell poles. IbpA-YFP, inclusion body protein A bound to yellow fluorescent protein.
(TIF)

S2 Fig Intracellular damage levels under light exposure. Intracellular damage at birth (*k*_*i*_) and division (*D*_*i*_) was estimated from growth parameters extracted for each population, based on individual doubling times. (A) The levels of damage inherited by new (blue) and old (red) daughters increased with the exposure to light excitation. An ANOVA revealed a significant effect of both exposure (*n* = 4,634 cells, F = 2,792.0, *p* < 0.001) and age (F = 968.4, *p* < 0.001) on inherited damage. Data are represented as mean ± SD. (B) Intracellular damage levels of populations at control conditions (reproduced from Fig 1E) or 3 s of light exposure. A significant difference was observed between *k*_*1*_ and *k*_*2*_ (paired one-tailed *t* test, *t* = 5.175, df = 69, *p* < 0.001) and between *D*_*1*_ and *D*_*2*_ (paired one-tailed *t* test, *t* = 5.304, df = 69, *p* < 0.001) in the 3-s treatment. Old daughters in the treatment were born with higher damage levels than in control (two-tailed *t* test, *t* = 32.408, df = 82.118, *p* <0.001). The difference *k*_*2*_ − *k*_*1*_ was significantly higher for control than treatment cells (two-tailed *t* test, *t* = 2.805, df = 80.995, *p* = 0.0063), an indication of higher symmetry in our 3-s treatment. Data are represented as mean ± SEM.
(TIF)

S1 Table Growth parameters of unstressed populations. Values of growth parameters Π (min), λ (min^−1^), and *a* obtained for wild-type populations.
(XLSX)

S2 Table Elongation rates of MG1655 populations exposed to extrinsic damage. Means and SDs of normalized elongation rates (min^−1^) measured for each level of light exposure, heat, or streptomycin, compared to its respective control.
(XLSX)

S3 Table Growth parameters of MG1655 exposed to phototoxic damage. Growth parameters λ (min^−1^) and *a* obtained for populations exposed to phototoxic damage, using Π from each respective control population.
(XLSX)

S4 Table Doubling time asymmetry of MG1655 exposed to extrinsic damage. Means and SDs of new and old daughter doubling times (min), along with pairwise comparison, for populations exposed to phototoxic damage, heat stress, or streptomycin. Pairs in which one daughter arrested division were excluded.
(XLSX)

S5 Table Variance partitioning in populations exposed to phototoxic damage. Partitioning of doubling time variances into stochastic ([V_N_ + V_o_] ÷ 2) and deterministic (D^2^ ÷ 4) components. Values presented as mean and 95% CIs. CI, confidence interval.
(XLSX)

S1 Data Doubling times of mothers, new siblings, and old siblings. Doubling times of unstressed wild-type populations as trios composed of a mother bacterium and its 2 daughters.
(XLSX)

S2 Data Doubling times of ClpB knockout mutants. Data for *ΔclpB* trios comprising a mother and its 2 daughters. ClpB, chaperone protein ClpB.
(XLSX)

S3 Data Doubling times of DnaK knockout mutants. Data for *ΔdnaK* trios comprising a mother and its 2 daughters. DnaK, chaperone protein DnaK.
(CSV)

S4 Data Doubling time of wild-type bacteria exposed to phototoxic damage. Data for wild-type trios comprising a mother and its 2 daughters, exposed to 70, 700, 1,000, 1,500, or 3,000 ms of light excitation.
(XLSX)

S5 Data Elongation rates of wild-type bacteria exposed to heat stress. Data for wild-type new and old daughters exposed to 38 °C, 40 °C, or 43 °C.
(XLSX)

S6 Data Elongation rates of wild-type bacteria exposed to streptomycin. Data for wild-type new and old daughters exposed to 2, 4, or 5 μg/ml streptomycin.
(XLSX)

S7 Data Data corresponding to summary statistics. Raw data used for Figs 1E, 2E, 3B, 4C, 4C, 4J, 5B and 5E, S1D, S1E, S2A and S2B Figs.
(XLSX)

## Abbreviations

CI: confidence interval
ClpB: chaperone protein ClpB
D: distance between distributions
DnaK: chaperone protein DnaK
IbpA: inclusion body protein A
LB: lysogeny broth
PDMS: polydimethylsiloxane
ROI: region of interest
V_N_: variance of new distributions
V_O_: variance of old distributions
V_T_: total variance
YFP: yellow fluorescent protein

## References

[1] López-Otín C, Blasco MA, Partridge L, Serrano M, Kroemer G. The hallmarks of aging. Cell. 2013;153: 1194–1217. 10.1016/j.cell.2013.05.039 23746838 PMC3836174

[2] Aravinthan A. Cellular senescence: a hitchhiker’s guide. Hum Cell. 2015;28: 51–64. 10.1007/s13577-015-0110-x 25690721

[3] Campisi J, d’Adda di Fagagna F. Cellular senescence: when bad things happen to good cells. Nat Rev Mol Cell Biol. 2007;8: 729–740. 10.1038/nrm2233 17667954

[4] Jeyapalan JC, Sedivy JM. Cellular senescence and organismal aging. Mech Ageing Dev. 2008;129: 467–474. 10.1016/j.mad.2008.04.001 18502472 PMC3297662

[5] Erjavec N, Cvijovic M, Klipp E, Nyström T. Selective benefits of damage partitioning in unicellular systems and its effects on aging. Proc Natl Acad Sci U S A. 2008;105: 18764–18769. 10.1073/pnas.0804550105 19020097 PMC2596250

[6] Laney SR, Olson RJ, Sosik HM. Diatoms favor their younger daughters. Limnol Oceanogr. 2012;57: 1572–1578. 10.4319/lo.2012.57.5.1572

[7] Rang CU, Peng AY, Chao L. Temporal dynamics of bacterial aging and rejuvenation. Curr Biol. 2011;21: 1813–6. 10.1016/j.cub.2011.09.018 22036179

[8] Stewart EJ, Madden R, Paul G, Taddei F. Aging and death in an organism that reproduces by morphologically symmetric division. PLoS Biol. 2005;3: e45 10.1371/journal.pbio.0030045 15685293 PMC546039

[9] Ksiazek K. Bacterial aging: From mechanistic basis to evolutionary perspective. Cell Mol Life Sci. 2010;67: 3131–3137. 10.1007/s00018-010-0417-4 20526791 PMC11115482

[10] Sabate R, De Groot NS, Ventura S. Protein folding and aggregation in bacteria. Cell Mol Life Sci. 2010;67: 2695–2715. 10.1007/s00018-010-0344-4 20358253 PMC11115605

[11] Lindner AB, Madden R, Demarez A, Stewart EJ, Taddei F. Asymmetric segregation of protein aggregates is associated with cellular aging and rejuvenation. Proc Natl Acad Sci U S A. 2008;105: 3076–81. 10.1073/pnas.0708931105 18287048 PMC2268587

[12] Winkler J, Seybert A, König L, Pruggnaller S, Haselmann U, Sourjik V, et al Quantitative and spatio-temporal features of protein aggregation in Escherichia coli and consequences on protein quality control and cellular ageing. EMBO J. 2010;29: 910–23. 10.1038/emboj.2009.412 20094032 PMC2837176

[13] Rokney A, Shagan M, Kessel M, Smith Y, Rosenshine I, Oppenheim AB. E. coli Transports Aggregated Proteins to the Poles by a Specific and Energy-Dependent Process. J Mol Biol. Elsevier Ltd; 2009;392: 589–601. 10.1016/j.jmb.2009.07.009 19596340

[14] Coquel AS, Jacob JP, Primet M, Demarez A, Dimiccoli M, Julou T, et al Localization of Protein Aggregation in Escherichia coli Is Governed by Diffusion and Nucleoid Macromolecular Crowding Effect. PLoS Comput Biol. 2013;9 10.1371/journal.pcbi.1003038 23633942 PMC3636022

[15] Ackermann M, Stearns SC, Jenal U. Senescence in a Bacterium with Asymmetric Division. Science. 2003;300: 1920 10.1126/science.1083532 12817142

[16] Rang CU, Peng AY, Poon AF, Chao L. Ageing in Escherichia coli requires damage by an extrinsic agent. Microbiology. 2012;158: 1553–9. 10.1099/mic.0.057240-0 22422756

[17] Proenca AM, Rang CU, Buetz C, Shi C, Chao L. Age structure landscapes emerge from the equilibrium between aging and rejuvenation in bacterial populations. Nat Commun. 2018;9: 3722 10.1038/s41467-018-06154-9 30213942 PMC6137065

[18] Chao L, Rang CU, Proenca AM, Chao JU. Asymmetrical Damage Partitioning in Bacteria: A Model for the Evolution of Stochasticity, Determinism, and Genetic Assimilation. PLoS Comput Biol. 2016;12: e1004700 10.1371/journal.pcbi.1004700 26761487 PMC4711911

[19] Fuentealba LC, Eivers E, Geissert D, Taelman V, De Robertis EM. Asymmetric mitosis: Unequal segregation of proteins destined for degradation. Proc Natl Acad Sci U S A. 2008;105: 7732–7737. 10.1073/pnas.0803027105 18511557 PMC2402384

[20] Moore DL, Pilz GA, Arauzo-Bravo MJ, Barral Y, Jessberger S. A mechanism for the segregation of age in mammalian neural stem cells. Science. 2015;349: 1334–1338. 10.1126/science.aac9868 26383951

[21] Bufalino MR, DeVeale B, van der Kooy D. The asymmetric segregation of damaged proteins is stem cell-type dependent. J Cell Biol. 2013;201: 523–530. 10.1083/jcb.201207052 23649805 PMC3653353

[22] Wang P, Robert L, Pelletier J, Dang WL, Taddei F, Wright A, et al Robust growth of Escherichia coli. Curr Biol. Elsevier Ltd; 2010;20: 1099–103. 10.1016/j.cub.2010.04.045 20537537 PMC2902570

[23] Chao L. A model for damage load and its implications for the evolution of bacterial aging. PLoS Genet. 2010;6: e1001076 10.1371/journal.pgen.1001076 20865171 PMC2928801

[24] Rang CU, Proenca AM, Buetz C, Shi C, Chao L. Minicells as a damage disposal mechanism in Escherichia coli. mSphere. 2018;3: e00428–18. 10.1128/mSphere.00428-18 30232168 PMC6147132

[25] Coelho M, Dereli A, Haese A, Kühn S, Malinovska L, Desantis ME, et al Fission yeast does not age under favorable conditions, but does so after stress. Curr Biol. 2013;23: 1844–1852. 10.1016/j.cub.2013.07.084 24035542 PMC4620659

[26] Govers SK, Mortier J, Adam A, Aertsen A. Protein aggregates encode epigenetic memory of stressful encounters in individual Escherichia coli cells. PLoS Biol. 2018;16: e2003853 10.1371/journal.pbio.2003853 30153247 PMC6112618

[27] Doyle SM, Wickner S. Hsp104 and ClpB: protein disaggregating machines. Trends Biochem Sci. 2009;34: 40–48. 10.1016/j.tibs.2008.09.010 19008106

[28] Dixit R, Cyr R. Cell damage and reactive oxygen species production induced by fluorescence microscopy: Effect on mitosis and guidelines for non-invasive fluorescence microscopy. Plant J. 2003;36: 280–290. 10.1046/j.1365-313X.2003.01868.x 14535891

[29] Gourmelon M, Cillard J, Pommepuy M. Visible light damage to Escherichia coli in seawater: oxidative stress hypothesis. J Appl Bacteriol. 1994;77: 105–112. 10.1111/j.1365-2672.1994.tb03051.x 7928776

[30] Godley BF, Shamsi FA, Liang F-Q, Jarrett SG, Davies S, Boulton M. Blue Light Induces Mitochondrial DNA Damage and Free Radical Production in Epithelial Cells. J Biol Chem. 2005;280: 21061–21066. 10.1074/jbc.M502194200 15797866

[31] Steiner UK, Lenart A, Ni M, Chen P, Song X, Taddei F, et al Two stochastic processes shape diverse senescence patterns in a single-cell organism. Evolution. 2019;73: 847–857. 30816556 10.1111/evo.13708

[32] Ni M, Decrulle AL, Fontaine F, Demarez A, Taddei F, Lindner AB. Pre-disposition and epigenetics govern variation in bacterial survival upon stress. PLoS Genet. 2012;8: e1003148 10.1371/journal.pgen.1003148 23284305 PMC3527273

[33] Rose MR. Evolutionary Biology of Aging. New York: Oxford University Press; 1991.

[34] Kirkwood TBL. Understanding ageing from an evolutionary perspective. J Intern Med. 2008;263: 117–127. 10.1111/j.1365-2796.2007.01901.x 18226090

[35] Baba T, Ara T, Hasegawa M, Takai Y, Okumura Y, Baba M, et al Construction of Escherichia coli K-12 in-frame, single-gene knockout mutants: The Keio collection. Mol Syst Biol. 2006;2 10.1038/msb4100050 16738554 PMC1681482

[36] Yang D, Jennings AD, Borrego E, Retterer ST, Männik J. Analysis of factors limiting bacterial growth in PDMS mother machine devices. Front Microbiol. 2018;9: 1–12. 29765371 10.3389/fmicb.2018.00871 PMC5938360

[37] Ducret A, Quardokus EM, Brun Y V. MicrobeJ, a tool for high throughput bacterial cell detection and quantitative analysis. Nat Microbiol. Nature Publishing Group; 2016;1 10.1038/nmicrobiol.2016.77 27572972 PMC5010025

