# Supplementary figures and images for: Cell aging preserves cellular immortality in the presence of lethal levels of damage
Source: PLoS Biol. 2019 May 23;17(5):e3000266. doi: 10.1371/journal.pbio.3000266 (PMC6532838; doi:10.1371/journal.pbio.3000266)

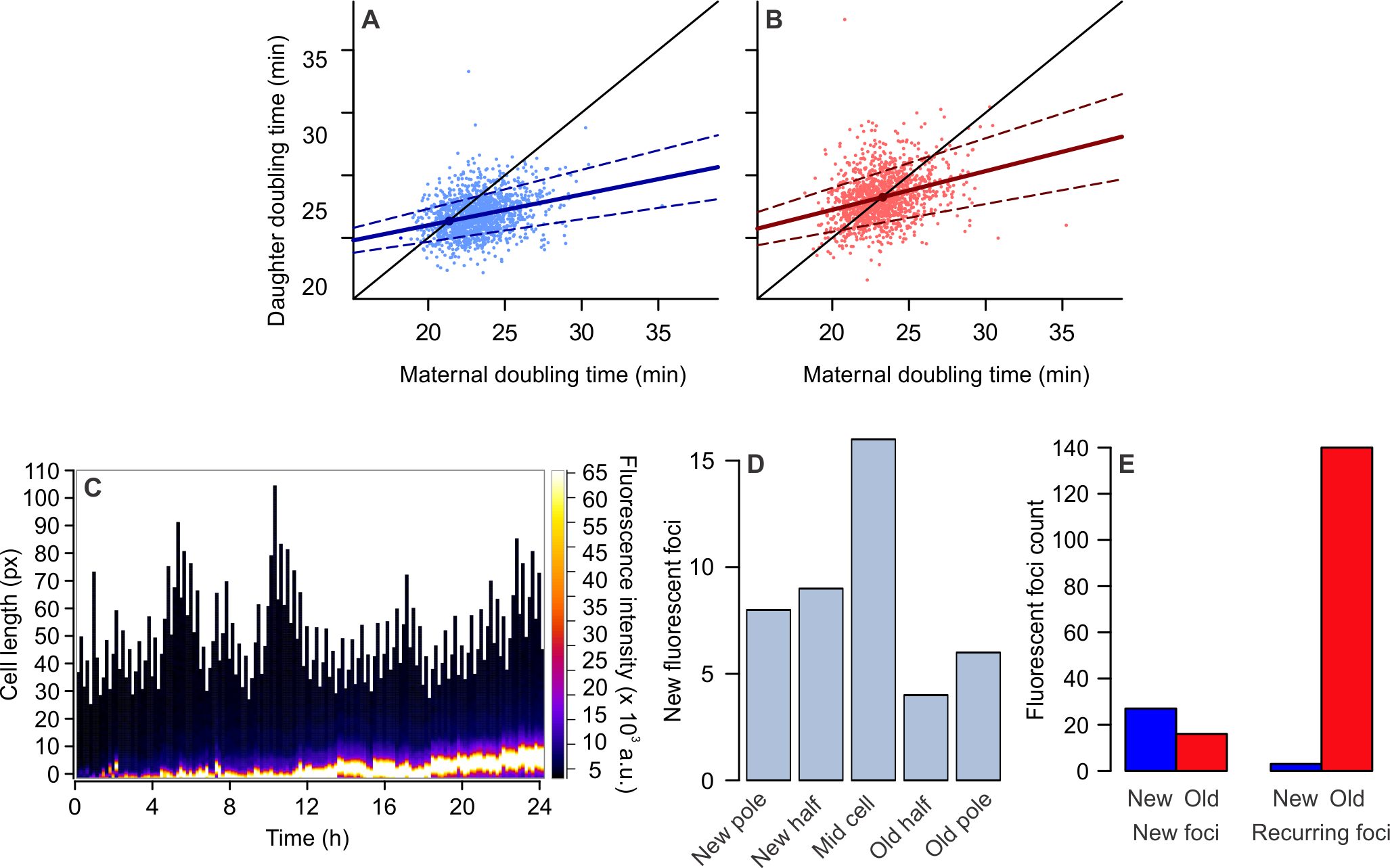

Supplement: S1 Fig — Growth stability in new and old lineages can be expressed by linear regressions between T0 and T1 (A, solid blue line) or T2 (B, solid red line). The intersect between regression lines and the identity line represents a point of stable equilibrium where doubling times converge. Due to the doubling time variance produced by stochasticity acting on the slopes (σ1), given by Ti = T0 × (a + σ1) + b, equilibria might be disrupted when a2 + σ12 ≥ 1. Dashed lines in (A) and (B) represent the maximum variation in regression lines obtained by the parameter σ1 acting on the slopes of our data, demonstrating that new and old lineages retain equilibrium in the presence of stochasticity. (C) Fluorescence profiles obtained in 10-min intervals for an old lineage, showing the anchoring of protein aggregates (IbpA-YFP) in the old pole over time. (D) Over the course of 194 cell divisions observed over 24-h imaging, we verified the first appearance of 43 protein aggregates. The cellular localization of these new fluorescent foci showed no bias for old poles. (E) The partitioning of new protein aggregates upon division showed higher inheritance by new daughters (62.79% of cell divisions, n = 43, χ2 = 4.651, df = 1, p = 0.031). However, old daughters inherited the majority of recurring aggregates (97.90% of cell divisions, n = 143, χ2 = 258.69, df = 1, p < 0.001) as these became anchored to old cell poles. IbpA-YFP, inclusion body protein A bound to yellow fluorescent protein. (TIF) [file pbio.3000266.s001.tif]

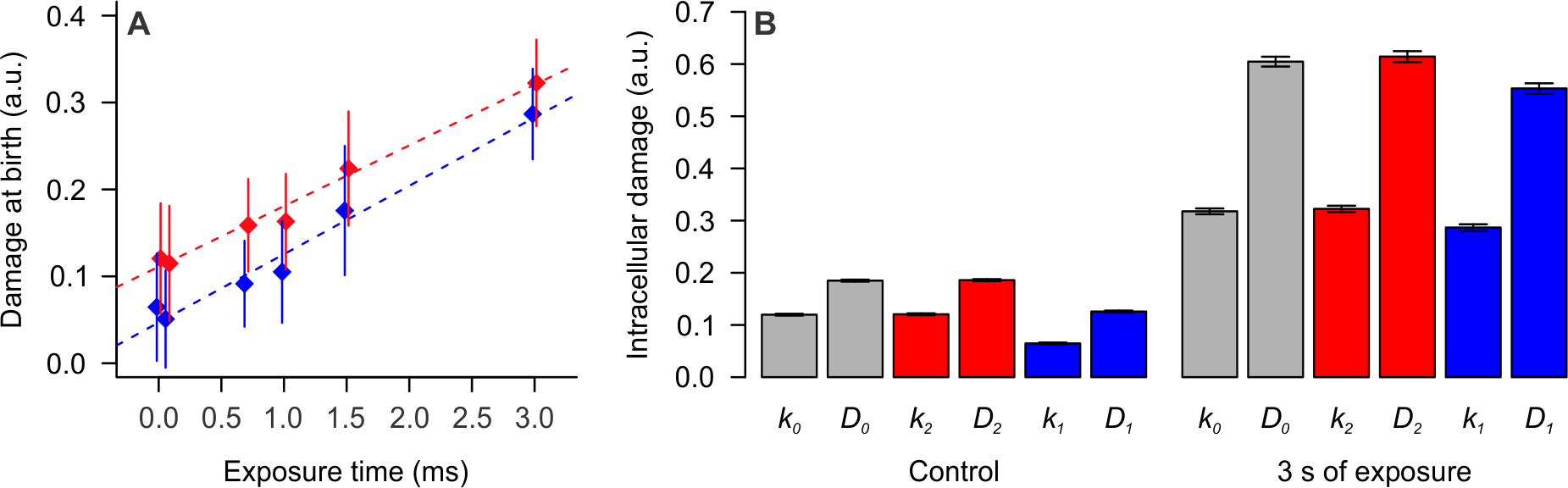

Supplement: S2 Fig — Intracellular damage at birth (ki) and division (Di) was estimated from growth parameters extracted for each population, based on individual doubling times. (A) The levels of damage inherited by new (blue) and old (red) daughters increased with the exposure to light excitation. An ANOVA revealed a significant effect of both exposure (n = 4,634 cells, F = 2,792.0, p < 0.001) and age (F = 968.4, p < 0.001) on inherited damage. Data are represented as mean ± SD. (B) Intracellular damage levels of populations at control conditions (reproduced from Fig 1E) or 3 s of light exposure. A significant difference was observed between k1 and k2 (paired one-tailed t test, t = 5.175, df = 69, p < 0.001) and between D1 and D2 (paired one-tailed t test, t = 5.304, df = 69, p < 0.001) in the 3-s treatment. Old daughters in the treatment were born with higher damage levels than in control (two-tailed t test, t = 32.408, df = 82.118, p <0.001). The difference k2 − k1 was significantly higher for control than treatment cells (two-tailed t test, t = 2.805, df = 80.995, p = 0.0063), an indication of higher symmetry in our 3-s treatment. Data are represented as mean ± SEM. (TIF) [file pbio.3000266.s002.tif]
